# Serum factors mediate changes in mitochondrial bioenergetics associated with diet and exercise interventions

**DOI:** 10.1007/s11357-023-00855-w

**Published:** 2023-06-27

**Authors:** Jenny L. Gonzalez-Armenta, Jaclyn Bergstrom, Jingyun Lee, Cristina M. Furdui, Barbara J. Nicklas, Anthony J. A. Molina

**Affiliations:** 1 Section On Gerontology and Geriatrics, Department of Internal Medicine, Wake Forest School of Medicine, Winston-Salem, NC USA; 2 Division of Geriatrics, Gerontology, and Palliative Care, Department of Medicine, University of California San Diego School of Medicine, 9500 Gilman Drive, MC 0665, La Jolla, CA 92093-0665 USA; 3 Proteomics and Metabolomics Shared Resource, Comprehensive Cancer Center, Wake Forest School of Medicine, Winston-Salem, NC USA; 4 Section On Molecular Medicine, Department of Internal Medicine, Wake Forest School of Medicine, Winston-Salem, NC USA

**Keywords:** Mitochondria, Bioenergetics, Resistance training, Caloric restriction, Older adults, Metabolomics

## Abstract

Mitochondrial improvements resulting from behavioral interventions, such as diet and exercise, are systemic and apparent across multiple tissues. Here, we test the hypothesis that factors present in serum, and therefore circulating throughout the body, can mediate changes in mitochondrial function in response to intervention. To investigate this, we used stored serum from a clinical trial comparing resistance training (RT) and RT plus caloric restriction (RT + CR) to examine effects of blood borne circulating factors on myoblasts in vitro. We report that exposure to dilute serum is sufficient to mediate bioenergetic benefits of these interventions. Additionally, serum-mediated bioenergetic changes can differentiate between interventions, recapitulate sex differences in bioenergetic responses, and is linked to improvements in physical function and inflammation. Using metabolomics, we identified circulating factors associated with changes in mitochondrial bioenergetics and the effects of interventions. This study provides new evidence that circulating factors play a role in the beneficial effects of interventions that improve healthspan among older adults. Understanding the factors that drive improvements in mitochondrial function is a key step towards predicting intervention outcomes and developing strategies to countermand systemic age-related bioenergetic decline.

**Supplementary Information:**

The online version contains supplementary material available at 10.1007/s11357-023-00855-w.

## Introduction

Mitochondrial dysfunction is among the cellular hallmarks of aging [44], and is characterized by reduced respiratory capacity, decreased mitochondrial enzyme activities, decreased mtDNA copy number, increased mtDNA mutations, increased ROS production, decreased mitochondrial content, and altered mitochondrial dynamics [5, 16, 18, 39, 48, 58, 64, 68, 72, 76]. To date, diet and exercise are among the most promising interventions to improve healthspan, and have been shown to target hallmarks of aging, including mitochondrial dysfunction [6, 24, 29, 37, 42, 50, 74]. Notably, age-related mitochondrial dysfunction and the improvements resulting from diet and exercise interventions are systemic and have been observed in a number of tissues, including the brain, heart, skeletal muscle, and liver [5, 12, 22, 27, 32].

Previous studies from our lab and others have utilized blood cells as reporters of systemic mitochondrial bioenergetic capacity in human studies [2, 9, 15, 38, 45, 54, 55, 69, 77-80, 84]. We have demonstrated that blood cell mitochondrial bioenergetics can recapitulate the bioenergetic capacity of the brain, skeletal muscle, and cardiac muscle and that increased circulating IL-6 is associated with decreased mitochondrial bioenergetic capacity in blood cells [77, 79, 80]. Taken together, these studies support the premise that blood cells are continuously exposed to circulating factors that mediate mitochondrial function across multiple tissues, and can therefore be used as reliable reporters of bioenergetic capacity. Utilizing the heterochronic parabiosis mouse model, we have demonstrated that circulating factors in the blood of older animals are sufficient to induce age-related changes in skeletal muscle mitochondrial structure and function [30]. Other groups have also shown that circulating factors play a role in the effects of aging and exercise interventions in a variety of tissues, including skeletal muscle, liver, brain, skin, and bone [3, 10, 17, 61, 66, 81]. These multiple lines of evidence suggest a potential role for blood-borne circulating factors in mediating the age-related bioenergetic decline and corresponding improvements from promising interventions such as diet and exercise.

In this study, we aimed to determine the effects of serum, collected from overweight and obese older adults, before and after resistance training or resistance training plus caloric restriction interventions, on muscle cells in vitro. We combined metabolomics and respirometry to identify circulating metabolites associated with changes in mitochondrial bioenergetics and response to resistance training and caloric restriction.

## Methods

### Parent study: I’M FIT

Samples utilized in this study were collected during the Improving Muscle for Functional Independence Trial (I’M FIT) (clinicaltrials.gov; NCT01049698). Briefly, I’M FIT was a 5-month, randomized controlled trial designed to determine whether CR enhances improvements in skeletal muscle function in response to RT in 126 older overweight and obese men and women. Participants were randomly assigned equally to a standardized, progressive RT intervention with CR (RT + CR) or without CR (RT) [56]. The study was approved by the Wake Forest School of Medicine Institutional Review Board, and all participants provided written informed consent to participate.

### Description of I’M FIT interventions

All participants in the study underwent 5 months of RT 3 days/week on weight-stack resistance machines (Cybex International Inc. and Nautilus Inc.) at the Wake Forest University Clinical Research Center exercise facility [56]. Briefly, the resistance training protocol was supervised by two exercise interventionists and included a gradual progression of weight and repetitions during the first month to allow familiarization with the equipment, minimize muscle soreness, and reduce injury potential. Subsequently, the training goal was to complete 3 sets of 10 repetitions for each exercise at 70% of their one-repetition maximum (1RM) for that specific exercise with 1RM testing every 4 weeks. Participants performed an initial 5-min warm-up by walking or cycling at a slow pace followed by light stretching and concluded each session with a 5-min cool-down and light stretching. The machines used were (1) leg press, (2) leg extension, (3) seated leg curl, (4) seated calf raise, (5) incline press, (6) compound row, (7) triceps press, and (8) biceps curl. Each participant recorded the weight lifted, the number of repetitions completed, and the number of sets completed for each exercise in a training log.

Participants assigned to RT only were instructed to follow a eucaloric diet, whereas those assigned to RT + CR underwent a dietary weight-loss intervention designed to elicit moderate weight loss (5–10%) over the 5-month period. Each participant was assigned a daily caloric intake, which was derived by subtracting 600 kcal from his or her estimated daily energy needs for weight maintenance. A maximum of 2 meal replacements per day (shakes and bars; Slim-Fast Inc.) that contained approximately 220 kcal with 7–10 g protein, 33–46 g carbohydrates, 1.5–5 g fat, and 2–5 g fiber were provided to participants for breakfast and lunch. Dinner and snack options were recommended by the RD per each participant’s daily caloric goals and tailored to allow for individual food preferences. Participants were asked to keep a diet log of all foods consumed, and the logs were monitored weekly by the RD to verify compliance with the weight-loss intervention.

### Measurements completed in the parent study

All assessments took place in the Geriatric Research Center of the Wake Forest School of Medicine J Paul Sticht Center on Aging by examiners blinded to participant treatment assignment [56]. Height, body mass, waist circumference, and hip circumference were measured. BMI was calculated as body mass divided by height squared. Waist-to-hip ratio was calculated as waist circumference divided by hip circumference. Grip strength was measured twice on each hand to the nearest kilogram by using an isometric Hydraulic Hand Dynamometer (Jamar), and the maximal value from the right hand was used in analyses. Mobility was assessed using the Mobility Assessment Tool–short form (MAT-sf) [62]. MAT-sf is a video-animated tool to assess self-perception of mobility that depicts a wooden mannequin performing a wide variety of physical activities (ranging from walking on level ground to carrying bags while climbing stairs) and a question about the participant’s ability to perform the task measured on a discrete scale with possible scores of 30–80 [63]. For 400-m walk time, the participant was instructed to complete the distance (10 laps on a flat indoor surface 20 m in length) as quickly as possible without running. Lower extremity function was assessed with the short physical performance battery (SPPB) [31], which consisted of a standing balance test, usual gait speed over a 4-m course, and time to complete 5 repeated chair rises with arms folded across the chest. Results from each of the three tests were scored from 0 (inability to perform the task to 4. The total SPPB score, which ranged from 0 (lowest function to 12 (highest function, was used for analyses. Maximal knee extensor strength (in Newton-meters [Nm] was measured with a dynamometer (Biodex Medical Systems Inc. at speeds of 608 and 2408/s with the participant sitting and hips and knees flexed at 90°. Participants were asked to extend the knee and push as hard as possible against the resistance pad. The strength of the right leg recorded as the peak torque (in Nm was used for analyses.

### Serum collection and storage

Blood samples were collected in serum separator tubes in the morning after an overnight fast at baseline and post-intervention (at least 24 h after the last acute bout of exercise). The blood was allowed to clot for 30–60 min, separated by centrifugation at 2000 × *g* for 10 min, divided into aliquots, and stored at − 80 °C. Of the original 126 participants of I’M FIT, 100 participants had pre- and post-intervention serum available for analysis.

### Inflammatory cytokines

IL-6 was measured by ELISA using high-sensitivity Quantikine Immunoassay kits from R&D Systems (Cat # SS600C). CRP was measured using high-sensitivity assays from Siemens Healthineers USA (Cat # LKCRP1) on an IMMULITE Automated Immunoassay System. To eliminate variability due to reagent conditions, all kits were pre-ordered having the same lot number for all reagents. All samples were measured in duplicate and the mean was used for data analyses. Samples with high variation (CV > 10%) were repeated. Commercial controls and an internal laboratory control were run on each 96-well plate.

### Cell culture, serum treatment, and metabolite treatment

C2C12 myoblast cells were obtained from ATCC (CRL-1772) and cultured in DMEM supplemented with 2 mM glutamine, 10% FBS, and 1% penicillin/streptomycin. Cells were seeded at a density of 1.5 × 10^6^ in T-75 culture flasks and were split every other day to avoid cultures becoming confluent.

For serum treatment, the experimental design was adapted from Cerqueira et al. [12]. In this study, C2C12 cells were seeded at a density of 15,000/well in a 96-well Seahorse culture plate and allowed to adhere overnight. Media was exchanged for serum-free media and 10% human serum was added to each well. Each participant’s pre- and post-serum was used for treatment in quadruplicate.

### Cell counting with Gen5

Cell images were obtained pre- and post-treatment with a Cytation 5 Cell Imaging Multi-Mode Reader (Biotek Instruments, Winooski, VT). These images were used to monitor cell health and proliferation during treatment. High-contrast brightfield images were used to count cells and were completed using Gen5 software (Biotek Instruments, Winooski, VT).

### Mitochondrial respirometry

Before each assay, media was exchanged for Seahorse XF assay media (Agilent, 103575) supplemented with 25 mM glucose, 2 mM glutamine, and 1 mM pyruvate. Additionally, brightfield images were obtained using Seahorse XF Imaging and Cell Counting Software (Agilent Technologies, Santa Clara, CA). Extracellular flux analysis was completed using a Seahorse XFe96 Analyzer (Agilent Technologies, Santa Clara, CA) with a standard injection protocol for a mitochondrial stress test: oligomycin (final concentration: 0.75 μM), FCCP (final concentration: 1 μM), and Antimycin-A/Rotenone (final concentration: 1 μM). Hoechst 3342 dye (final concentration: 6 μM) was injected at the end of the assay and incubated at 37 °C for 30 min before fluorescence imaging in the XF Imaging and Cell Counting Software for normalization to cell count. Seahorse assay parameters (basal, ATP-linked, leak, max, and spare respiratory capacity) were corrected for non-mitochondrial respiration and calculated as previously described [23].

### Respirometry data exclusion criteria

Respirometry was completed with samples from 100 participants that had completed the intervention and had pre- and post-serum available. We applied a strict data quality control process prior to any statistical analyses. Wells were excluded from Seahorse analysis if (1) OCR measurements were negative or below 10 pmol/min, (2) oxygen level diverged more than 20 mmHg from background wells (typically a level of ~ 150 mmHg), (3) poor nuclear staining due to insufficient cell number, (4) only one or two wells were remaining per sample (i.e., only one well would not be included in the analysis for a pre-intervention sample), or (5) lack of paired pre- or post-intervention data (only complete pre- and post-intervention pairs were included in the analysis). After application of these pre-determined exclusion criteria, 33 participants that completed the RT intervention and 35 participants that completed the RT + CR intervention were included in this study, a total of 68 participants.

### Metabolomic analysis

LC-MS-grade water, acetonitrile, methanol, and formic acid were purchased from ThermoFisher Scientific (Waltham, MA, USA). 2-(N-morpholino)ethanesulfonic acid (MES) and ammonium formate were purchased from Sigma-Aldrich (St. Louis, MO, USA).

Fifty microliters of I’M FIT serum (total *n* = 68, RT = 33, RT + CR = 35) was spiked with 10 µL of MES solution which was freshly prepared in water (20 ng/mL). Metabolites were extracted by adding 200 µL of cold methanol and incubated on ice for 30 min. The supernatant was taken after centrifugation at 18,000 × *g* for 5 min and was dried under vacuum. The residue was reconstituted in 5% methanol for LC-MS analysis.

The LC-MS consisted of a Q Exactive HF hybrid quadrupole-Orbitrap mass spectrometer (Thermo Scientific, Waltham, MA, USA) and a Vanquish UHPLC system (Thermo Scientific, Waltham, MA, USA). Samples were analyzed on two different columns, a Hypersil GOLD pentafluorophenyl (PFP) column (2.1 × 100 mm, 1.9 µm; Thermo Scientific, Waltham, MA, USA) and an Accucore Vanquish C18 + (2.1 × 100 mm, 1.5 µm; Thermo Scientific, Waltham, MA, USA) column. A linear gradient was employed for chromatographic separation using 100% water (mobile phase A) and 90% acetonitrile (mobile phase B), both of which contained 0.1% formic acid and 10 mM ammonium formate. For the PFP column, the mobile phase flow rate was 0.25 mL/min and the gradient began at 2% B that was held for 2 min, and then increased to 98% B at 8 min. This percentage was held for 2 min before being decreased to 2% B at 10.1 min and then held at that final percentage until 13 min. For the C18 column, the mobile phase flow rate was 0.20 mL/min and the gradient began at 0% B which was held for 3 min, then increased to 95% B at 6 min. This percentage was held for 1 min before being decreased to 0% B at 7.1 min and then held at that final percentage until 10 min. Data was acquired by collecting full mass spectra (MS1) using polarity switching (positive/negative) at a resolution of 150 K.

To identify metabolites, peak features were detected and integrated by the MSMLS Discovery software (IROA Technologies LLC, Sea Girt, NJ, USA) in combination with in-house compound libraries prepared using a Mass Spectrometry Metabolite Library (Sigma, St. Louis, MO, USA). To eliminate redundancy in compound identification, the most abundant ion in each metabolite was selected, which was then normalized to the total ion current (TIC) for relative quantification.

### Statistical analysis

Statistical significance between groups (pre- and post-intervention or RT and RT + CR) was evaluated by paired two-tailed Student’s *t*-tests. Significance between groups was defined as *p* ≤ 0.05. In addition, we examined the statistical differences between groups for post-intervention outcome values using an ANCOVA and a model adjusted for age, sex, and the baseline value for the outcome.

Spearman correlations were assessed between changes in bioenergetic parameters and physical function and inflammatory cytokines with both groups combined, separated by intervention group, and separated by sex. Partial correlations were adjusted for age, BMI, sex, baseline physical function, and baseline bioenergetics. Significance was defined as *p* ≤ 0.05. Analysis was performed using SAS Enterprise Guide 7.12 (SAS Institute Inc., Cary, NC, USA).

To classify participants as negative responders, non-responders, and positive responders based on change in serum-mediated maximal respiration, we modified the method described by Dankel et. al. [20], to account for random error associated with measuring outcomes in exercise interventions. Briefly, we used the RT group to calculate a 75% confidence interval (CI) to account for 75% of the random error. For negative responders, we included participants who had a change in maximal respiration below the 75% CI, and for positive responders, we included participants who had a change in maximal respiration above the 75% CI. A summary of these parameters is shown in Supplemental Table 5.

Analysis of the metabolomic data was performed using MetaboAnalyst 5.0 [14]. Spearman correlations were assessed between change in bioenergetic parameters and fold change of metabolites. Significance was defined as *p* ≤ 0.05. Analysis was performed using SAS Enterprise Guide 7.12 (SAS Institute Inc., Cary, NC, USA).

## Results

### Participant demographics and characteristics at baseline

Age, number of female participants, number of white individuals, body mass, height, BMI, waist circumference, hip circumference, and waist-to-hip ratio for the participants included in this ancillary study are summarized in Table 1. There were no significant differences identified between the two intervention groups, RT or RT + CR.

**Table 1:** Participant demographics and physical characteristics (mean ± SD, *n* [%]) at baseline

	RT (*n* = 33)	RT + CR (*n* = 35)	*p*-Value
Age, years	69.4 ± 3.0	69.0 ± 3.2	0.63
Female, *n* (%)	15 (45)	14 (40)	0.65
White, *n* (%)	27 (82)	31 (89)	0.51
Body mass, kg	87.3 ± 15.0	86.7 ± 12.6	0.85
Height, cm	167.5 ± 12.0	168.2 ± 9.9	0.78
BMI, kg/cm^2^	30.9 ± 2.5	30.5 ± 2.4	0.47
Waist, cm	95.7 ± 9.8	96.5 ± 10.0	0.72
Hip, cm	108.6 ± 7.1	108.1 ± 8.0	0.80
Waist-to-hip ratio	0.88 ± 0.09	0.90 ± 0.09	0.59

### Serum-mediated bioenergetics and the effects of intervention

**Fig. 1:**
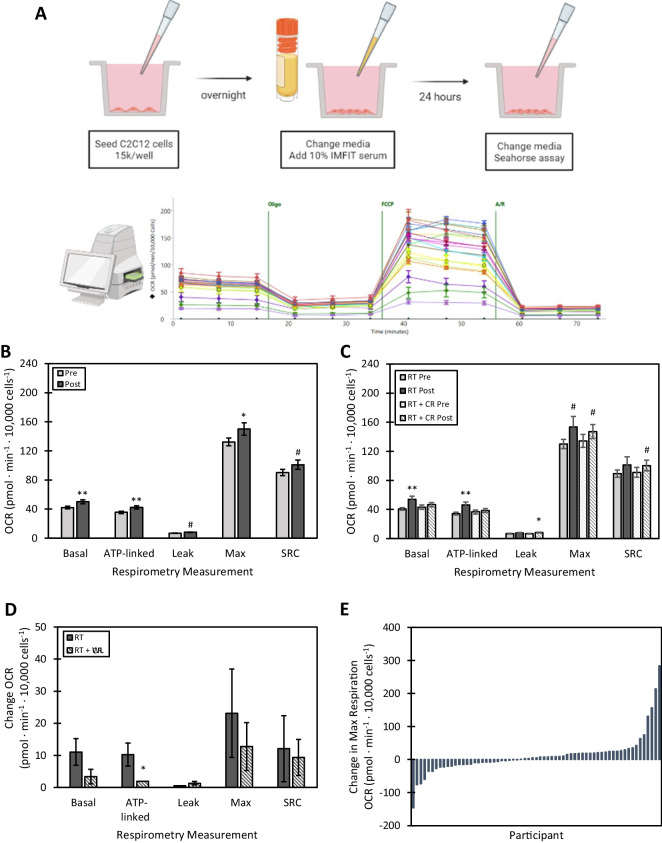
Myoblasts exposed to dilute serum collected before and after intervention exhibit bioenergetic changes associated with RT and RT + CR. **A** Overall design of serum-mediated bioenergetic experiments. **B** Overall effect of intervention on serum-mediated bioenergetics with both intervention groups combined. Data are represented as mean ± SEM. Difference from pre, *p*-value: ** < 0.01, * < 0.05, ^#^ < 0.10. **C** Effect of intervention (RT vs RT + CR) on serum-mediated bioenergetics. Data are represented as mean ± SEM. Difference from pre, *p*-value: ** < 0.01, * < 0.05, ^#^ < 0.10. **D** Effect of intervention (RT vs RT + CR) on change in serum-mediated bioenergetics. Data are represented as mean ± SEM. Difference between groups, *p*-value: ** < 0.01, * < 0.05, ^#^ < 0.10. **E** Change in max respiration in all participants

The experimental design and mitochondrial respirometry results are summarized in Fig. 1. The overall effect of intervention on serum-mediated bioenergetics with both intervention groups combined is presented in Fig. 1B. With both intervention groups combined, post-intervention serum-mediated bioenergetics were significantly increased compared to pre-intervention serum-mediated bioenergetics as reported by basal, ATP-linked, max, and spare respiratory capacity. The effects of each intervention, RT or RT + CR, on serum-mediated bioenergetics are shown in Fig. 1C and the change in OCR from each intervention is shown in Fig. 1D. With RT, post-intervention serum-mediated bioenergetics were significantly increased compared to pre-intervention serum-mediated bioenergetics as measured by basal and ATP-linked and there was a trend for increased max respiration. With RT + CR, there were trends for increased post-intervention serum-mediated bioenergetics compared to pre-intervention serum-mediated bioenergetics as measured by max and spare respiratory capacity. The change in max respiration in all participants is shown in Fig. 1E, demonstrating the heterogeneity of bioenergetic changes with intervention. The unadjusted mitochondrial respiration and physical function measurements at baseline and changes with intervention are summarized in Supplemental Table 1.

Supplemental Fig. 1 presents the change in cell count after serum treatment (Supplemental Fig. 1A and B) and the observed sex differences in these cell counts (Supplemental Fig. 1C and D). Cell proliferation upon overnight incubation with participant serum varied depending on intervention group and sex. In particular, RT and female serum appeared to increase overnight proliferation, though these differences were not statistically significant.

**Fig. 2:**
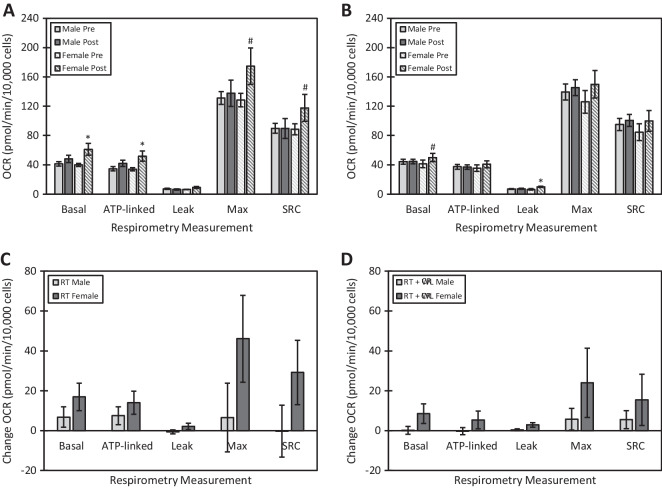
Serum-mediated bioenergetic changes in response to intervention exhibit sex differences. **A** Effect of RT intervention on serum-mediated bioenergetics by sex. Data are represented as mean ± SEM. Difference from pre, *p*-value: * < 0.05, ^#^ < 0.10. **B** Effect of RT + CR intervention on serum-mediated bioenergetics by sex. Data are represented as mean ± SEM. Difference from pre, *p*-value: * < 0.05, ^#^ < 0.10. **C** Effect of sex (male vs female) on change in serum-mediated bioenergetics in the RT group. Data are represented as mean ± SEM. **D** Effect of sex (male vs female) on change in serum-mediated bioenergetics in the RT + CR group. Data are represented as mean ± SEM

Observed sex differences in serum-mediated bioenergetics are shown in Fig. 2. Sex differences in OCR pre- and post-intervention are shown for RT and RT + CR in Fig. 2A and B, respectively. Serum from female participants who completed the RT intervention significantly increased post-intervention basal and ATP-linked respiration and had trends for increased maximal respiration and spare respiratory capacity when compared to pre-intervention. Sex differences in change in OCR in response to intervention are shown for RT and RT + CR in Fig. 2C and D, respectively. Serum from female participants who completed the RT + CR intervention had a trend for increased post-intervention basal respiration and significantly increased leak respiration when compared to pre-intervention. There were no statistically significant differences between pre- and post-intervention serum-mediated bioenergetics with serum from male participants from either intervention, RT or RT + CR.

### Correlations between change in serum-mediated bioenergetics and change in physical function and inflammatory cytokines

Spearman correlations were used to assess relationships between bioenergetic parameters, physical function, and inflammatory cytokines and are shown in Supplemental Table 2 (all participants combined), Supplemental Table 3 (separated by intervention group), and Supplemental Table 4 (separated by sex). We observed associations between improved mitochondrial bioenergetics and improved physical function and markers of inflammation. In particular, the relationships with physical function (MAT-sf and gait speed) were stronger in the RT group and the relationships with inflammation (Il-6 and CRP) were stronger in the RT + CR group. We also observed sex differences in these correlations. The improvements in MAT-sf and CRP were more strongly correlated with bioenergetics in male versus female participants. Female participant bioenergetic parameters were more strongly correlated with improvements in 400-m walk time and IL-6 when compared to male participants.

**Fig. 3:**
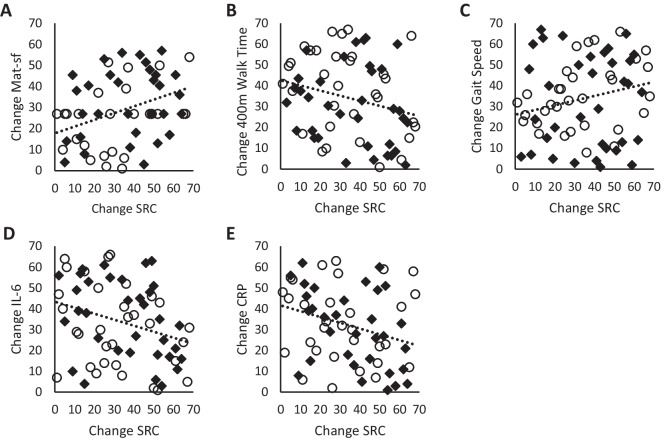
Changes in serum-mediated max respiration are correlated with changes in physical function and inflammatory cytokines. **A** Spearman correlation between change in spare respiratory capacity and change in mobility as assessed by MAT-sf (*R* = 0.372, *p* = 0.004). **B** Spearman correlation between change in spare respiratory capacity and change in 400-m walk time (*R* =  − 0.246, *p* = 0.04). **C** Spearman correlation between change in spare respiratory capacity and change in gait speed (*R* = 0.224, *p* = 0.07). **D** Spearman correlation between change in spare respiratory capacity and change in IL-6 (*R* =  − 0.290, *p* = 0.02). **E** Spearman correlation between change in spare respiratory capacity and change in CRP (*R* =  − 0.294, *p* = 0.02)

Regression plots illustrating the relationships between change in serum-mediated SRC and physical function are shown in Fig. 3A–E. Change in serum-mediated SRC was significantly and positively correlated with change in MAT-sf (Fig. 3F; *R* = 0.372, *p* = 0.004) and significantly and negatively correlated with change in 400-m walk time (Fig. 3G; *R* =  − 0.246, *p* = 0.04); a trend for a positive correlation with change in gait speed (Fig. 3H; *R* = 0.224, *p* = 0.07) was significantly and negatively correlated with change in IL-6 (Fig. 3I; *R* =  − 0.290, *p* = 0.02) and was significantly and negatively correlated with change in CRP (Fig. 3J; *R* =  − 0.294, *p* = 0.02).

### Metabolomic analysis reveals candidate serum metabolites associated with bioenergetic changes in response to diet and exercise interventions

**Fig. 4:**
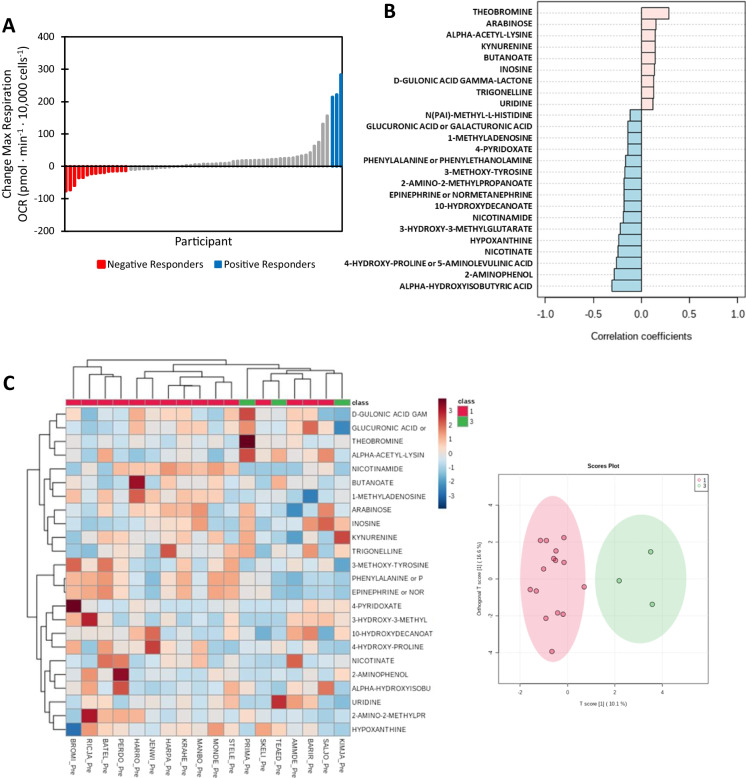
Metabolomic analysis reveals potential candidate metabolites that could mediate muscle bioenergetic changes in response to diet and exercise interventions. **A** Using change in maximal respiration, we classified participants as negative responders, non-responders, and positive responders for metabolomic analysis. **B** Pattern analysis reveals 25 potential metabolites that are either positively or negatively associated with the pattern of negative responders to non-responders to positive responders. **C** Heat map illustrating relationships of fold change of top 25 metabolites and responder status and score plot from orthogonal-partial least squares analysis illustrating the complete separation of negative and positive responders based on fold change of metabolites

We sought to determine if changes in serum metabolites are related the heterogeneity of serum-mediated bioenergetic response to intervention. To do this, we first classified the participants as negative responders, non-responders, and positive responders based on the change in serum-mediated maximal respiration (shown in Supplemental Table 5) and these are shown in Fig. 4A. We used pattern analysis to determine which metabolites were either positively or negatively associated with the pattern of negative responders to non-responders to positive responders. These metabolites are shown in Fig. 4B. A heat map illustrating relationships of fold change of these top 25 metabolites and negative and positive responders and the score plot from orthogonal-partial least squares analysis illustrating the separation of negative and positive responders are shown in Fig. 4C.

### Correlations between change in serum-mediated bioenergetics and fold change of candidate metabolites

**Fig. 5:**
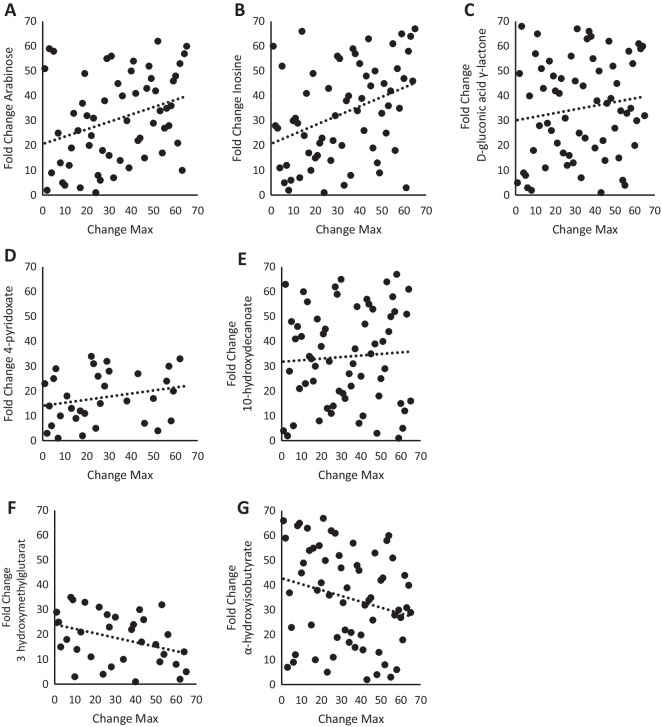
Changes in serum-mediated max respiration are correlated with fold changes of identified candidate metabolites. **A** Spearman correlation between change in max respiration and fold change of arabinose (*R* = 0.320, *p* = 0.01). **B** Spearman correlation between change in max respiration and fold change of inosine (*R* = 0.367, *p* = 0.003). **C** Spearman correlation between change in max respiration and fold change of D-gluconic acid γ-lactone (*R* = 0.270, *p* = 0.08). **D** Spearman correlation between change in max respiration and fold change of 4-pyridoxate (*R* = 0.275, *p* = 0.11). **E** Spearman correlation between change in max respiration and fold change of 10-hydroxydecanoate (*R* = 0.060, *p* = 0.64). **F** Spearman correlation between change in max respiration and fold change of 3-hydroxymethylglutarate (*R* =  − 0.359, *p* = 0.04). **G** Spearman correlation between change in max respiration and fold change of α-hydroxyisobutyrate (*R* =  − 0.242, *p* = 0.05)

Spearman correlations were used to assess relationships between bioenergetic parameters and the top 25 metabolites identified. Significant relationships are summarized in Table 2. Regression plots illustrating the relationships between change in serum-mediated max respiration and fold change of metabolites are shown in Fig. 5. Change in serum-mediated max respiration was significantly and positively correlated with fold change of arabinose (Fig. 5A; *R* = 0.320, *p* = 0.01), was significantly and positively correlated with fold change of inosine (Fig. 5B; *R* = 0.367, *p* = 0.003), had a trend for a positive association with fold change of d-gluconic acid γ-lactone (Fig. 5C; *R* = 0.270, *p* = 0.08), was not significantly associated with fold change of 4-pyridoxate (Fig. 5D; *R* = 0.275, *p* = 0.11), was not associated with fold change of 10-hydroxydecanoate (Fig. 5E; *R* = 0.060, *p* = 0.64), was significantly and negatively correlated with fold change of 3-hydroxymethylglutarate (Fig. 5F; *R* =  − 0.359, *p* = 0.04), and had a trend for a negative correlation with fold change of α-hydroxyisobutyrate (Fig. 5G; *R* =  − 0.242, *p* = 0.05).

**Table 2:** Spearman correlations between change in mitochondrial respiration measurements and fold change of metabolites

	Basal	ATP-linked	Leak	Max	SRC
Arabinose	0.282*	0.313*	0.080	0.320*	0.315*
Inosine	0.260*	0.278*	0.087	0.367**	0.304*
D-gluconic acid γ-lactone	0.062	0.038	0.229^#^	0.270*	0.330*
4-Pyridoxate	0.401*	0.386*	0.295	0.275	0.168
10-Hydroxydecanoate	− 0.201	− 0.242^#^	0.061	0.060	0.078
3-Hydroxymethylglutarate	− 0.455*	− 0.448*	− 0.101	− 0.359*	− 0.334
α-Hydroxyisobutyrate	− 0.331*	− 0.380**	0.001	− 0.242^#^	− 0.155

^**^ < 0.01, * < 0.05, ^#^ < 0.10

## Discussion

In this study, we aimed to determine the effects of human serum collected before and after diet and exercise interventions on myoblasts in vitro and to identify circulating metabolites associated with these changes. We began by examining the overall effect of intervention on serum-mediated bioenergetics and found that post-intervention serum significantly increased basal, ATP-linked, and maximal mitochondrial respiration when compared to pre-intervention. These results are consistent with observations of increased skeletal muscle mitochondrial function after exercise interventions in older adults [47, 49, 50, 59, 60, 74]. Importantly, these results provide striking new evidence that non-cellular factors present in serum can mediate improvements in key measures of mitochondrial function associated with intervention.

Next, we examined the effect of intervention group, either RT or RT + CR, on serum-mediated bioenergetics. We found that post-RT serum significantly increased basal and ATP-linked mitochondrial respiration and had a trend for increased maximal mitochondrial respiration, while post-RT + CR serum had trends for increased maximal mitochondrial respiration and spare respiratory capacity. Previous studies examining the effect of RT on mitochondria report increased mitochondrial function as measured by phosphocreatine recovery time [47] and high-resolution respirometry of permeabilized skeletal muscle fibers [57, 60]. Studies examining the effect of CR alone on skeletal muscle mitochondria found no changes in mitochondrial content [75] or mitochondrial function as measured by phosphocreatine recovery time [71]. Exercise and CR are rarely combined in studies examining mitochondrial function, but others have reported that aerobic exercise increased mitochondrial content and mitochondrial enzyme activities involved in electron transport and fatty acid oxidation, while caloric restriction to induce weight loss did not [51]. Our results provide new data indicating that the heterogeneous bioenergetic effects of intervention on muscle mitochondrial function can be mediated by changes in non-cellular circulating factors.

Our results indicate that improvements in serum-mediated bioenergetics after RT + CR serum treatment are less robust compared to RT alone, suggesting that CR may blunt the bioenergetic effects of RT with serum-mediated bioenergetics. Alternatively, RT may alleviate some of the reduced mitochondrial bioenergetics associated with CR. Both of these possibilities are consistent with observations that CR decreases overall energy expenditure and increases work efficiency [33], RT prevents a substantial amount of lean mass loss associated with CR [67], and that lean mass is positively associated with mitochondrial bioenergetics in monocytes [7]. Therefore, it is possible that CR blunts some of the positive effects of RT, while RT alleviates some of the negative effects of CR on mitochondrial bioenergetics.

Notably, we observed a high level of diversity in the range of responses to intervention with serum-mediated bioenergetics. This is best exemplified by examining the change in maximal respiration across all participants. We identified three groups of responders: negative responders, non-responders, and positive responders. While there is debate in the literature about whether there are true non-responders or if some of these observations could be attributed to measurement variability that is not adequately controlled for [20, 70], this observation is also consistent with a number of previous studies that reported heterogeneous responses to a variety of exercise interventions [4, 8, 36, 40, 57, 65]. Indeed, this pattern of heterogeneous response in terms of physical function was also observed in the participants from I’M FIT, the parent study for the samples utilized in this study [13].

Data on serum-mediated bioenergetics presented in this manuscript are all normalized to cell count. Cell proliferation upon overnight incubation with participant serum varied depending on intervention group and sex. Others have noted that serum source affects cell growth, proliferation, and migration of cultured cells as well as spheroid formation [34, 35]. These findings highlight the importance of normalization in this study and future studies.

We observed binary sex differences in serum-mediated bioenergetics. Serum from female participants in the RT group significantly increased basal and ATP-linked mitochondrial respiration and had trends for increased maximal respiration and spare respiratory capacity. We did not observe any significant increases in the male participants. These findings are consistent with observations with high-resolution respirometry that females have greater intrinsic mitochondrial respiratory rates [11] and exhibit decreased ADP sensitivity and increased sensitivity to malonyl-CoA-mediated respiratory inhibition in skeletal muscle compared to men [52]. Furthermore, these observations are consistent with other studies, including sex differences in fiber type and fuel utilization during exercise (reviewed in [1]. Importantly, this is the first study to illustrate that sex differences in response to interventions can be mediated by serum factors alone.

We found significant correlations between improvements in serum-mediated bioenergetics and improvements in physical function, measured by MAT-sf, 400-m walk time, and gait speed, and improvements in inflammation, measured by IL-6 and CRP. These results are consistent with cross-sectional studies completed in a subset of I’M FIT participants, where higher blood cell bioenergetics was associated with faster gait speed, knee extensor strength, and grip strength as well as lower levels of IL-6 [77, 78]. Taken together, this suggests that improvements in mitochondrial bioenergetics, physical function, and inflammation in older adults are systemic and mediated by blood-borne circulating factors.

Metabolomic analyses identified circulating factors associated with changes in bioenergetic capacity. Our analyses revealed 25 circulating factors that were positively or negatively associated with the pattern of responses that we observed in the change in serum-mediated maximal respiration. In particular, we identified 3 metabolites that were significantly and positively associated with serum-mediated maximal respiration (arabinose, inosine, and d-gluconic acid γ-lactone) and 2 metabolites that were significantly and negatively associated with serum-mediated maximal respiration (3-hydroxymethylglutarate and α-hydroxisobutyrate). Of these, inosine, 3-hydroxymethyl-3-glutarate, and α-hydroxisobutyrate have been associated with metabolism, exercise performance, and age-related conditions.

Inosine is a purine nucleoside produced by the catabolism of adenosine and has been examined for potential use in improving exercise performance [46, 73, 83], epilepsy [41], multiple sclerosis [53], and Parkinson’s disease [82] due to potential neuroprotective properties shown in rats [43, 85].

3-Hydroxymethylglutarate is an off-product that is a result of the incomplete conversion of 3-hydroxy-3-methylglutaryl-CoA to acetyl-CoA and acetoacetate by defective or inefficient 3-hydroxy-3-methylglutaryl-CoA lyase. 3-Hydroxymethyl-3-glutarate accumulates in the mitochondria and high levels in urine have been associated with inborn errors of metabolism [21, 25]. Our results suggest that, in older adults, resistance training and caloric restriction either leads to the reduction of 3-hydroxymethyl-3-glutarate directly or reduces the negative effects of 3-hydroxymethyl-3-glutarate on mitochondrial bioenergetics through mechanisms that can be explored in future studies.α-Hydroxisobutyrate is an organic acid derived from α-ketobutyrate that is produced by amino acid catabolism and glutathione anabolism [28]. α-Hydroxisobutyrate has been suggested as a potential early biomarker for insulin resistance and glucose intolerance [26, 28]. In particular, α-hydroxisobutyrate has been associated with increased insulin resistance and in vitro treatment of pancreatic β-cells with α-hydroxisobutyrate inhibited glucose-mediated insulin secretion [26]. We found a strong relationship between increasing concentrations of α-hydroxisobutyrate and decreased serum-mediated bioenergetics. Taken together, this suggests that α-hydroxisobutyrate may play a key role in the development of insulin resistance, both through the inhibition of insulin secretion in β-cells and the decrease in skeletal muscle mitochondrial respiration observed here, and the improvements in insulin sensitivity that are observed with RT and CR interventions.

Though they were not significantly correlated with serum-mediated bioenergetics, it was interesting that kynurenine, nicotinamide, and nicotinate were among the top 25 circulating factors associated with changes in mitochondrial bioenergetics. These metabolites are important in NAD biosynthesis and recycling pathways [19] and suggest that alterations in NAD metabolism could be involved in mediating mitochondrial metabolism as well.

The major strengths of this study include the leveraging of a randomized controlled clinical trial and the utilization of an approach that could be readily applied to other clinical studies. The approach described here can be utilized to examine the bioenergetic effects of various conditions and activities using stored biorepository samples. Furthermore, combining our bioenergetic profiling approach with omic technologies that assess the contents of human serum, e.g., metabolites, proteins, and lipids, can reveal candidate factors that may influence mitochondrial function systemically. Potential limitations of this study include the sole focus on overweight and obese older adults. We are unable to evaluate whether our findings extend to other age groups and to individuals who are not overweight, or if they are specific to the demographic enrolled by the parent trial. Moreover, while the identities of circulating factors we identified to be associated with the bioenergetic effects of serum provide information about the metabolic pathways leading to their production, we are unable to determine their source, i.e., the cells and tissues responsible for production, based on the analysis of serum alone.

In this study, we report that serum and the circulating factors therein are sufficient to recapitulate the effects of a RT or RT + CR intervention in vitro, including differentiating between the interventions, demonstrating sex differences in bioenergetic response to intervention, and is linked to key improvements in physical function and inflammation. We also identified circulating metabolites that are associated with changes in mitochondrial bioenergetics. This study further supports a growing body of evidence that circulating factors are not only important for the aging process, but in interventions to improve healthspan as well. Future studies may be able to determine the specific effects of candidate circulating factors on mitochondrial function across multiple tissue types. Furthermore, it remains to be determined how specific factors in serum can exert effects targeting skeletal muscle and other tissues of interest in vivo. For example, circulating factors may exert effects on vascular endothelial cells that impact transport to target tissues.

### Supplementary Information

Below is the link to the electronic supplementary material.

Supplementary file1 (DOCX 210 KB)

## References

[1] Ansdell P, Thomas K, Hicks KM, Hunter SK, Howatson G, Goodall S (2020). Physiological sex differences affect the integrative response to exercise: acute and chronic implications. Exp Physiol.

[2] Avila C, Huang RJ, Stevens MV, Aponte AM, Tripodi D, Kim KY, Sack MN (2012). Platelet mitochondrial dysfunction is evident in type 2 diabetes in association with modifications of mitochondrial anti-oxidant stress proteins. Exp Clin Endocrinol Diabetes.

[3] Baht GS, Silkstone D, Vi L, Nadesan P, Amani Y, Whetstone H, Wei Q, Alman BA (2015). Exposure to a youthful circulaton rejuvenates bone repair through modulation of β-catenin. Nat Commun.

[4] Baird JF, Motl RW (2019). Response heterogeneity with exercise training and physical activity interventions among persons with multiple sclerosis. Neurorehabil Neural Repair.

[5] Barazzoni R, Short KR, Nair KS (2000). Effects of aging on mitochondrial DNA copy number and cytochrome c oxidase gene expression in rat skeletal muscle, liver, and heart. J Biol Chem.

[6] Barbosa de Queiroz K, Honorato-Sampaio K, Rossoni Júnior JV, Andrade Leal D, Pinto ABG, Kappes-Becker L, Evangelista EA, Guerra-Sá R (2017). Physical activity prevents alterations in mitochondrial ultrastructure and glucometabolic parameters in a high-sugar diet model. PLoS ONE.

[7] Bellissimo M, Fleischer C, Tran P, Hao L, Reiter D, Smith M, Boebinger S, Wells G, Jones D, Ziegler T et al. Mitochondrial bioenergetic metabolism is associated with total body composition and influenced by normal weight obesity (P21-039-19). Curr Dev Nutr. 2019;3.

[8] Bouchard C, An P, Rice T, Skinner JS, Wilmore JH, Gagnon J, Pérusse L, Leon AS, Rao DC (1999). Familial aggregation of VO(2max) response to exercise training: results from the HERITAGE Family Study. J Appl Physiol.

[9] Braganza A, Corey CG, Santanasto AJ, Distefano G, Coen PM, Glynn NW, Nouraie SM, Goodpaster BH, Newman AB, Shiva S. Platelet bioenergetics correlate with muscle energetics and are altered in older adults. JCI Insight. 2019;5. 10.1172/jci.insight.128248. 10.1172/jci.insight.128248 PMC6629251 31120438

[10] Bu X-L, Xiang Y, Jin W-S, Wang J, Shen L-L, Huang Z-L, Zhang K, Liu Y-H, Zeng F, Liu J-H (2018). Blood-derived amyloid-β protein induces Alzheimer’s disease pathologies. Mol Psychiatry.

[11] Cardinale DA, Larsen FJ, Schiffer TA, Morales-Alamo D, Ekblom B, Calbet JAL, Holmberg H-C, Boushel R (2018). Superior intrinsic mitochondrial respiration in women than in men. Front Physiol.

[12] Cerqueira FM, Chausse B, Baranovski BM, Liesa M, Lewis EC, Shirihai OS, Kowaltowski AJ (2016). Diluted serum from calorie-restricted animals promotes mitochondrial β-cell adaptations and protect against glucolipotoxicity. FEBS J.

[13] Chmelo EA, Crotts CI, Newman JC, Brinkley TE, Lyles MF, Leng X, Marsh AP, Nicklas BJ (2015). Heterogeneity of physical function responses to exercise training in older adults. J Am Geriatr Soc.

[14] Chong J, Wishart DS, Xia J (2019). Using MetaboAnalyst 4.0 for comprehensive and integrative metabolomics data analysis. Curr Protoc Bioinforma.

[15] Chou C-H, Fu T-C, Tsai H-H, Hsu C-C, Wang C-H, Wang J-S (2019). High-intensity interval training enhances mitochondrial bioenergetics of platelets in patients with heart failure. Int J Cardiol.

[16] Coggan AR, Spina RJ, Rogers MA, King DS, Brown M, Nemeth PM, Holloszy JO (1990). Histochemical and enzymatic characteristics of skeletal muscle in master athletes. J Appl Physiol.

[17] Conboy IM, Rando TA (2012). Heterochronic parabiosis for the study of the effects of aging on stem cells and their niches. Cell Cycle.

[18] Conley KE, Jubrias SA, Esselman PC (2000). Oxidative capacity and ageing in human muscle. J Physiol.

[19] Covarrubias AJ, Perrone R, Grozio A, Verdin E (2021). NAD^+^ metabolism and its roles in cellular processes during ageing. Nat Rev Mol Cell Biol.

[20] Dankel SJ, Loenneke JP (2020). A method to stop analyzing random error and start analyzing differential responders to exercise. Sports Med.

[21] Dena R, Fabbro M, Rigoni F (1978). Formation and utilization of 3-hydroxy-3-methylglutarate in liver mitochondria of starved and streptozotocin-diabetic rats. Biochem J.

[22] Desler C, Hansen TL, Frederiksen JB, Marcker ML, Singh KK, Juel Rasmussen L (2012). Is there a link between mitochondrial reserve respiratory capacity and aging?. J Aging Res.

[23] Divakaruni AS, Rogers GW, Murphy AN. Measuring mitochondrial function in permeabilized cells using the seahorse XF analyzer or a clark‐type oxygen electrode. Curr Protoc Toxicol. 2014;60(1):25.2. 1–2. 16. 10.1002/0471140856.tx2502s60 24865646

[24] Drake JC, Wilson RJ, Yan Z (2016). Molecular mechanisms for mitochondrial adaptation to exercise training in skeletal muscle. FASEB J.

[25] Fernandes CG, Rodrigues MDN, Seminotti B, Colín-González AL, Santamaria A, Quincozes-Santos A, Wajner M (2016). Induction of a proinflammatory response in cortical astrocytes by the major metabolites accumulating in HMG-CoA lyase deficiency: the role of ERK signaling pathway in cytokine release. Mol Neurobiol.

[26] Ferrannini E, Natali A, Camastra S, Nannipieri M, Mari A, Adam K-P, Milburn MV, Kastenmüller G, Adamski J, Tuomi T (2013). Early metabolic markers of the development of dysglycemia and type 2 diabetes and their physiological significance. Diabetes.

[27] Forni MF, Peloggia J, Braga TT, Chinchilla JEO, Shinohara J, Navas CA, Camara NOS, Kowaltowski AJ (2017). Caloric restriction promotes structural and metabolic changes in the skin. Cell Rep.

[28] Gall WE, Beebe K, Lawton KA, Adam K.-P, Mitchell MW, Nakhle PJ, Ryals JA, Milburn MV, Nannipieri M, Camastra S et al. α-Hydroxybutyrate is an early biomarker of insulin resistance and glucose intolerance in a nondiabetic population. PLoS One. 2010;5(5):e10883. 10.1371/journal.pone.0010883 PMC2878333 20526369

[29] Garatachea N, Pareja-Galeano H, Sanchis-Gomar F, Santos-Lozano A, Fiuza-Luces C, Morán M, Emanuele E, Joyner MJ, Lucia A (2015). Exercise attenuates the major hallmarks of aging. Rejuvenation Res.

[30] Gonzalez-Armenta JL, Li N, Lee R-L, Lu B, Molina AJA (2021). Heterochronic parabiosis: old blood induces changes in mitochondrial structure and function of young mice. J Gerontol A Biol Sci Med Sci.

[31] Guralnik JM, Simonsick EM, Ferrucci L, Glynn RJ, Berkman LF, Blazer DG, Scherr PA, Wallace RB (1994). A short physical performance battery assessing lower extremity function: association with self-reported disability and prediction of mortality and nursing home admission. J Gerontol.

[32] Gusdon AM, Callio J, Distefano G, O’Doherty RM, Goodpaster BH, Coen PM, Chu CT (2017). Exercise increases mitochondrial complex I activity and DRP1 expression in the brains of aged mice. Exp Gerontol.

[33] Hames KC, Coen PM, King WC, Anthony SJ, Stefanovic-Racic M, Toledo FGS, Brown J, Helbling N, Dubé JJ, DeLany JP (2016). Resting and exercise energy metabolism in weight-reduced adults with severe obesity. Obesity (Silver Spring).

[34] Heger JI, Froehlich K, Pastuschek J, Schmidt A, Baer C, Mrowka R, Backsch C, Schleußner E, Markert UR, Schmidt A (2018). Human serum alters cell culture behavior and improves spheroid formation in comparison to fetal bovine serum. Exp Cell Res.

[35] Hennig B, Boissonneault GA, Glauert HP (1989). Effects of serum type on growth and permeability properties of cultured endothelial cells. Exp Cell Res.

[36] Hubal MJ, Gordish-Dressman H, Thompson PD, Price TB, Hoffman EP, Angelopoulos TJ, Gordon PM, Moyna NM, Pescatello LS, Visich PS (2005). Variability in muscle size and strength gain after unilateral resistance training. Med Sci Sports Exerc.

[37] Hyatt HW, Kephart WC, Holland AM, Mumford P, Mobley CB, Lowery RP, Roberts MD, Wilson JM, Kavazis AN (2016). A ketogenic diet in rodents elicits improved mitochondrial adaptations in response to resistance exercise training compared to an isocaloric Western diet. Front Physiol.

[38] Japiassú AM, Santiago APSA, da d’Avila JCP, Garcia-Souza LF, Galina A, Castro Faria-Neto HC, Bozza FA, Oliveira MF (2011). Bioenergetic failure of human peripheral blood monocytes in patients with septic shock is mediated by reduced F1Fo adenosine-5′-triphosphate synthase activity. Crit Care Med.

[39] Johannsen DL, Conley KE, Bajpeyi S, Punyanitya M, Gallagher D, Zhang Z, Covington J, Smith SR, Ravussin E (2012). Ectopic lipid accumulation and reduced glucose tolerance in elderly adults are accompanied by altered skeletal muscle mitochondrial activity. J Clin Endocrinol Metab.

[40] Kohrt WM, Malley MT, Coggan AR, Spina RJ, Ogawa T, Ehsani AA, Bourey RE, Martin WH, Holloszy JO (1991). Effects of gender, age, and fitness level on response of VO2max to training in 60–71 yr olds. J Appl Physiol.

[41] Kovacs Z, Kekesi KA, Juhasz G, Barna J, Heja L, Lakatos R, Dobolyi A (2015). Non-adenosine nucleoside inosine, guanosine and uridine as promising antiepileptic drugs: a summary of current literature. Mini Rev Med Chem.

[42] Lanza IR, Nair KS (2009). Muscle mitochondrial changes with aging and exercise. Am J Clin Nutr.

[43] Liu F, You S-W, Yao L-P, Liu H-L, Jiao X-Y, Shi M, Zhao Q-B, Ju G (2006). Secondary degeneration reduced by inosine after spinal cord injury in rats. Spinal Cord.

[44] López-Otín C, Blasco MA, Partridge L, Serrano M, Kroemer G (2013). The hallmarks of aging. Cell.

[45] Mahapatra G, Smith SC, Hughes TM, Wagner B, Maldjian JA, Freedman BI, Molina AJA (2018). Blood-based bioenergetic profiling is related to differences in brain morphology in African Americans with type 2 diabetes. Clin Sci (Lond).

[46] McNaughton L, Dalton B, Tarr J (1999). Inosine supplementation has no effect on aerobic or anaerobic cycling performance. Int J Sport Nutr.

[47] Meex RCR, Schrauwen-Hinderling VB, Moonen-Kornips E, Schaart G, Mensink M, Phielix E, van de Weijer T, Sels J-P, Schrauwen P, Hesselink MKC (2010). Restoration of muscle mitochondrial function and metabolic flexibility in type 2 diabetes by exercise training is paralleled by increased myocellular fat storage and improved insulin sensitivity. Diabetes.

[48] Melov S, Shoffner JM, Kaufman A, Wallace DC (1995). Marked increase in the number and variety of mitochondrial DNA rearrangements in aging human skeletal muscle. Nucleic Acids Res.

[49] Menshikova EV, Ritov VB, Toledo FGS, Ferrell RE, Goodpaster BH, Kelley DE (2005). Effects of weight loss and physical activity on skeletal muscle mitochondrial function in obesity. Am J Physiol Endocrinol Metab.

[50] Menshikova EV, Ritov VB, Fairfull L, Ferrell RE, Kelley DE, Goodpaster BH (2006). Effects of exercise on mitochondrial content and function in aging human skeletal muscle. J Gerontol A Biol Sci Med Sci.

[51] Menshikova EV, Ritov VB, Dube JJ, Amati F, Stefanovic-Racic M, Toledo FGS, Coen PM, Goodpaster BH (2018). Calorie restriction-induced weight loss and exercise have differential effects on skeletal muscle mitochondria despite similar effects on insulin sensitivity. J Gerontol: Ser A.

[52] Miotto PM, McGlory C, Holloway TM, Phillips SM, Holloway GP (2018). Sex differences in mitochondrial respiratory function in human skeletal muscle. Am J Physiol-Regul Integr Comp Physiol.

[53] Muñoz García D, Midaglia L, Martinez Vilela J, Marín Sánchez M, López González FJ, Arias Gómez M, Dapena Bolaño D, Iglesias Castañón A, Alonso Alonso M, Romero López J (2015). Associated inosine to interferon: results of a clinical trial in multiple sclerosis. Acta Neurol Scand.

[54] Nguyen QL, Corey C, White P, Watson A, Gladwin MT, Simon MA, Shiva S (2017). Platelets from pulmonary hypertension patients show increased mitochondrial reserve capacity. JCI Insight.

[55] Nguyen QL, Wang Y, Helbling N, Simon MA, Shiva S (2019). Alterations in platelet bioenergetics in Group 2 PH-HFpEF patients. PLoS ONE.

[56] Nicklas BJ, Chmelo E, Delbono O, Carr JJ, Lyles MF, Marsh AP (2015). Effects of resistance training with and without caloric restriction on physical function and mobility in overweight and obese older adults: a randomized controlled trial. Am J Clin Nutr.

[57] Pesta D, Hoppel F, Macek C, Messner H, Faulhaber M, Kobel C, Parson W, Burtscher M, Schocke M, Gnaiger E (2011). Similar qualitative and quantitative changes of mitochondrial respiration following strength and endurance training in normoxia and hypoxia in sedentary humans. Am J Physiol Regul Integr Comp Physiol.

[58] Petersen KF, Befroy D, Dufour S, Dziura J, Ariyan C, Rothman DL, DiPietro L, Cline GW, Shulman GI (2003). Mitochondrial dysfunction in the elderly: possible role in insulin resistance. Science.

[59] Phielix E, Meex R, Moonen-Kornips E, Hesselink MKC, Schrauwen P (2010). Exercise training increases mitochondrial content and ex vivo mitochondrial function similarly in patients with type 2 diabetes and in control individuals. Diabetologia.

[60] Porter C, Reidy PT, Bhattarai N, Sidossis LS, Rasmussen BB (2015). Resistance exercise training alters mitochondrial function in human skeletal muscle. Med Sci Sports Exerc.

[61] Rebo J, Mehdipour M, Gathwala R, Causey K, Liu Y, Conboy MJ, Conboy IM (2016). A single heterochronic blood exchange reveals rapid inhibition of multiple tissues by old blood. Nat Commun.

[62] Rejeski WJ, Ip EH, Marsh AP, Barnard RT (2010). Development and validation of a video-animated tool for assessing mobility. J Gerontol: Ser A.

[63] Rejeski WJ, Rushing J, Guralnik JM, Ip EH, King AC, Manini TM, Marsh AP, McDermott MM, Fielding RA, Newman AB (2015). The MAT-sf: identifying risk for major mobility disability. J Gerontol: Ser A.

[64] Rooyackers OE, Adey DB, Ades PA, Nair KS (1996). Effect of age on in vivo rates of mitochondrial protein synthesis in human skeletal muscle. Proc Natl Acad Sci U S A.

[65] Ross R, Goodpaster BH, Koch LG, Sarzynski MA, Kohrt WM, Johannsen NM, Skinner JS, Castro A, Irving BA, Noland RC (2019). Precision exercise medicine: understanding exercise response variability. Br J Sports Med.

[66] Salpeter S, Khalaileh A, Weinberg-Corem N, Ziv O, Glaser B, Dor Y (2013). Systemic regulation of the age-related decline of pancreatic β-cell replication. Diabetes.

[67] Sardeli A, Komatsu T, Mori M, Gaspari A, Chacon-Mikahil M (2018). Resistance training prevents muscle loss induced by caloric restriction in obese elderly individuals: a systematic review and meta-analysis. Nutrients.

[68] Short KR, Bigelow ML, Kahl J, Singh R, Coenen-Schimke J, Raghavakaimal S, Nair KS (2005). Decline in skeletal muscle mitochondrial function with aging in humans. Proc Natl Acad Sci U S A.

[69] Sjövall F, Morota S, Hansson MJ, Friberg H, Gnaiger E, Elmér E (2010). Temporal increase of platelet mitochondrial respiration is negatively associated with clinical outcome in patients with sepsis. Crit Care.

[70] Sparks LM (2017). Exercise training response heterogeneity: physiological and molecular insights. Diabetologia.

[71] Sparks LM, Redman LM, Conley KE, Harper M-E, Yi F, Hodges A, Eroshkin A, Costford SR, Gabriel ME, Shook C (2016). Effects of 12 months of caloric restriction on muscle mitochondrial function in healthy individuals. J Clin Endocrinol Metab.

[72] Srivastava S. The mitochondrial basis of aging and age-related disorders. Genes (Basel). 2017;8. 10.3390/genes8120398 PMC5748716 29257072

[73] Starling RD, Trappe TA, Short KR, Sheffield-Moore M, Jozsi AC, Fink WJ, Costill DL (1996). Effect of inosine supplementation on aerobic and anaerobic cycling performance. Med Sci Sports Exerc.

[74] Toledo FGS, Goodpaster BH (2013). The role of weight loss and exercise in correcting skeletal muscle mitochondrial abnormalities in obesity, diabetes and aging. Mol Cell Endocrinol.

[75] Toledo FGS, Menshikova EV, Azuma K, Radiková Z, Kelley CA, Ritov VB, Kelley DE (2008). Mitochondrial capacity in skeletal muscle is not stimulated by weight loss despite increases in insulin action and decreases in intramyocellular lipid content. Diabetes.

[76] Tonkonogi M, Fernström M, Walsh B, Ji LL, Rooyackers O, Hammarqvist F, Wernerman J, Sahlin K (2003). Reduced oxidative power but unchanged antioxidative capacity in skeletal muscle from aged humans. Pflugers Arch.

[77] Tyrrell DJ, Bharadwaj MS, Van Horn CG, Marsh AP, Nicklas BJ, Molina AJA (2015). Blood-cell bioenergetics are associated with physical function and inflammation in overweight/obese older adults. Exp Gerontol.

[78] Tyrrell DJ, Bharadwaj MS, Van Horn CG, Kritchevsky SB, Nicklas BJ, Molina AJA (2015). Respirometric profiling of muscle mitochondria and blood cells are associated with differences in gait speed among community-dwelling older adults. J Gerontol A Biol Sci Med Sci.

[79] Tyrrell DJ, Bharadwaj MS, Jorgensen MJ, Register TC, Molina AJA (2016). Blood cell respirometry is associated with skeletal and cardiac muscle bioenergetics: implications for a minimally invasive biomarker of mitochondrial health. Redox Biol.

[80] Tyrrell DJ, Bharadwaj MS, Jorgensen MJ, Register TC, Shively C, Andrews RN, Neth B, Keene CD, Mintz A, Craft S (2017). Blood-based bioenergetic profiling reflects differences in brain bioenergetics and metabolism. Oxid Med Cell Longev.

[81] Villeda SA, Luo J, Mosher KI, Zou B, Britschgi M, Bieri G, Stan TM, Fainberg N, Ding Z, Eggel A (2011). The ageing systemic milieu negatively regulates neurogenesis and cognitive function. Nature.

[82] Watanabe H, Hattori T, Kume A, Misu K, Ito T, Koike Y, Johnson TA, Kamitsuji S, Kamatani N, Sobue G (2020). Improved Parkinsons disease motor score in a single-arm open-label trial of febuxostat and inosine. Medicine (Baltimore).

[83] Williams MH, Kreider RB, Hunter DW, Somma CT, Shall LM, Woodhouse ML, Rokitski L (1990). Effect of inosine supplementation on 3-mile treadmill run performance and VO_2_ peak. Med Sci Sports Exerc.

[84] Willig AL, Kramer PA, Chacko BK, Darley-Usmar VM, Heath SL, Overton ET (2017). Monocyte bioenergetic function is associated with body composition in virologically suppressed HIV-infected women. Redox Biol.

[85] Zai L, Ferrari C, Subbaiah S, Havton LA, Coppola G, Strittmatter S, Irwin N, Geschwind D, Benowitz LI (2009). Inosine alters gene expression and axonal projections in neurons contralateral to a cortical infarct and improves skilled use of the impaired limb. J Neurosci.

