# Supplementary material for: Serum factors mediate changes in mitochondrial bioenergetics associated with diet and exercise interventions
Source: GeroScience. 2023 Jun 27;46(1):349–65. doi: 10.1007/s11357-023-00855-w (PMC10828137; doi:10.1007/s11357-023-00855-w)
Supplement: Supplementary file 1 — Supplementary file1 (DOCX 210 KB) [file 11357_2023_855_MOESM1_ESM.docx]

**
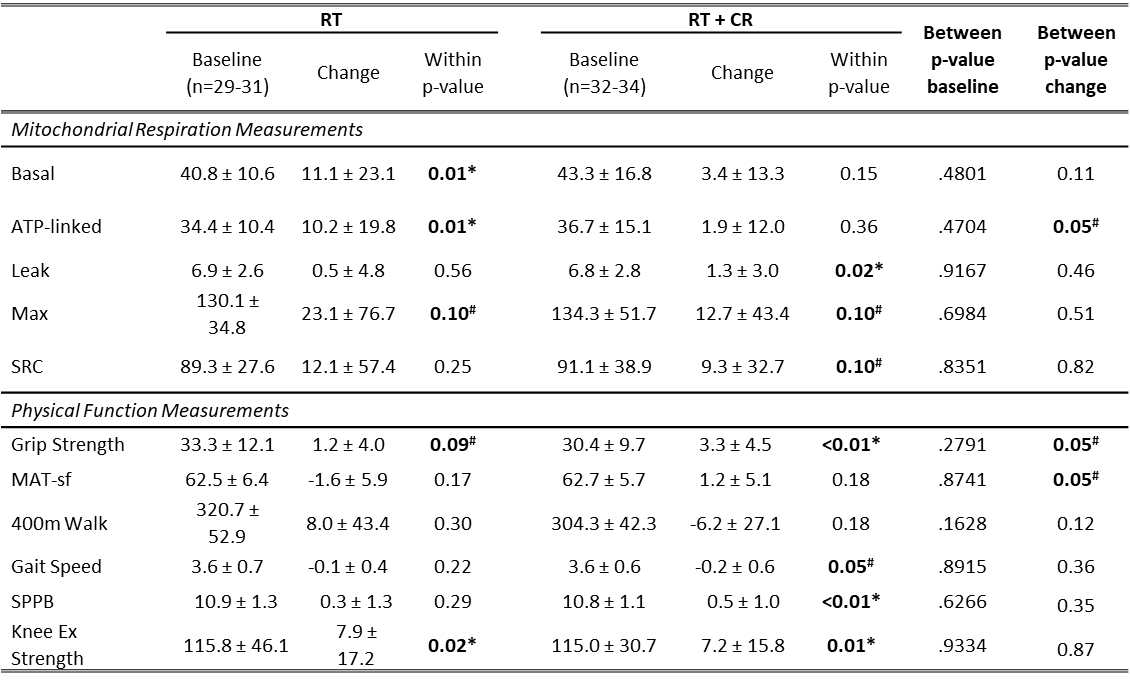
Supplemental Table 1.** Unadjusted mitochondrial respiration and physical function measurements at baseline and changes with intervention (** < 0.01, * < 0.05, ^#^ < 0.10)

**
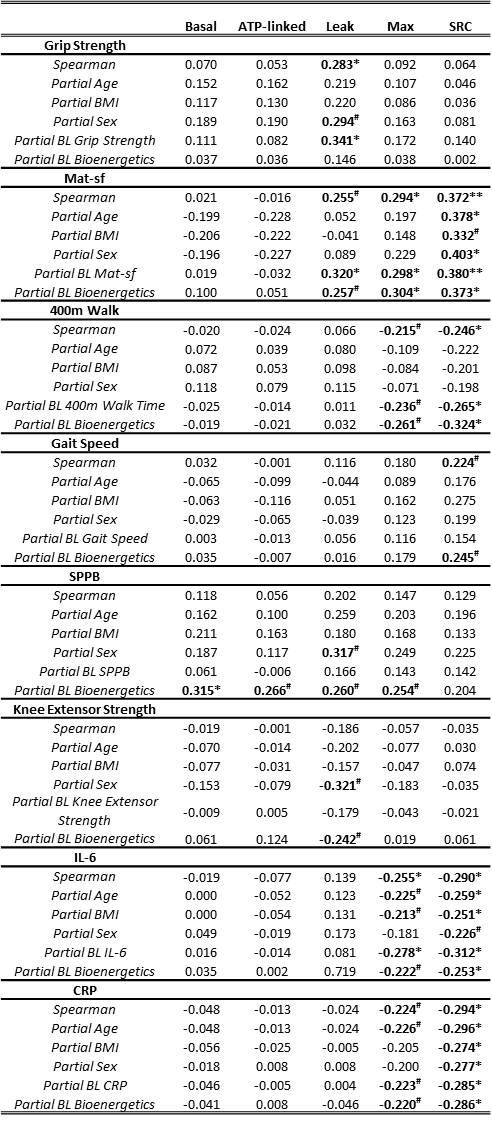
Supplemental Table 2.** Correlations between change in mitochondrial respiration measurements and change in physical function and inflammatory cytokines. All participants (** < 0.01, * < 0.05, ^#^ < 0.10)

**
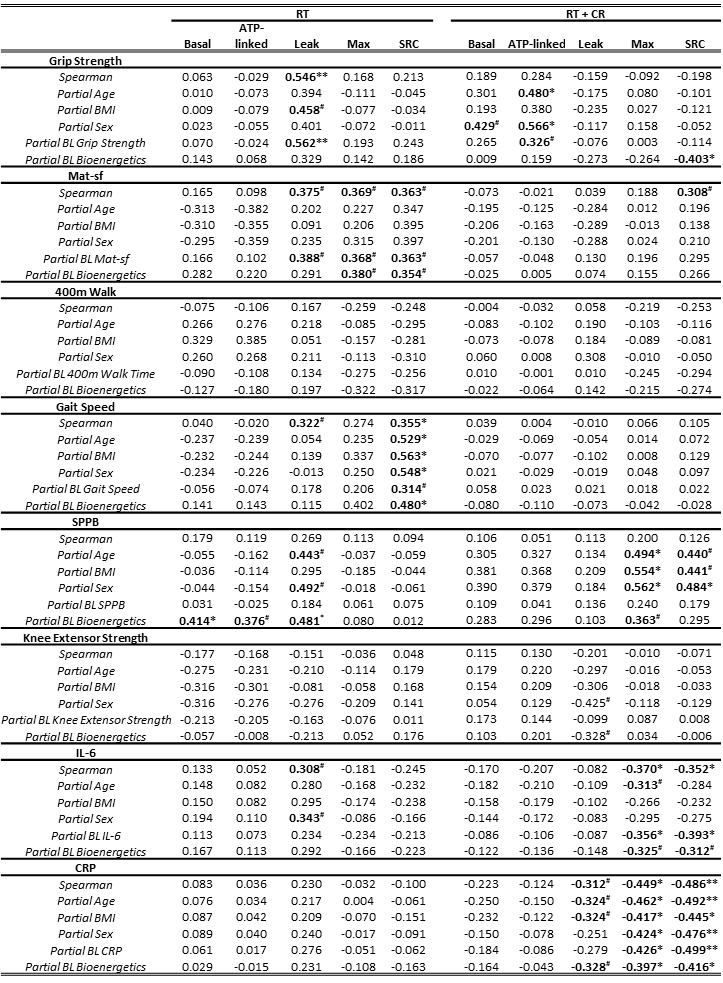
Supplemental Table 3.** Spearman correlations between change in mitochondrial respiration measurements and change in physical function and inflammatory cytokines. Analysis by intervention group (** < 0.01, * < 0.05, ^#^ < 0.10)

**Supplemental Table 4.** Spearman correlations between change in mitochondrial respiration measurements and change in physical function and inflammatory cytokines. Analysis by sex (** < 0.01, * < 0.05, ^#^ < 0.10)

**
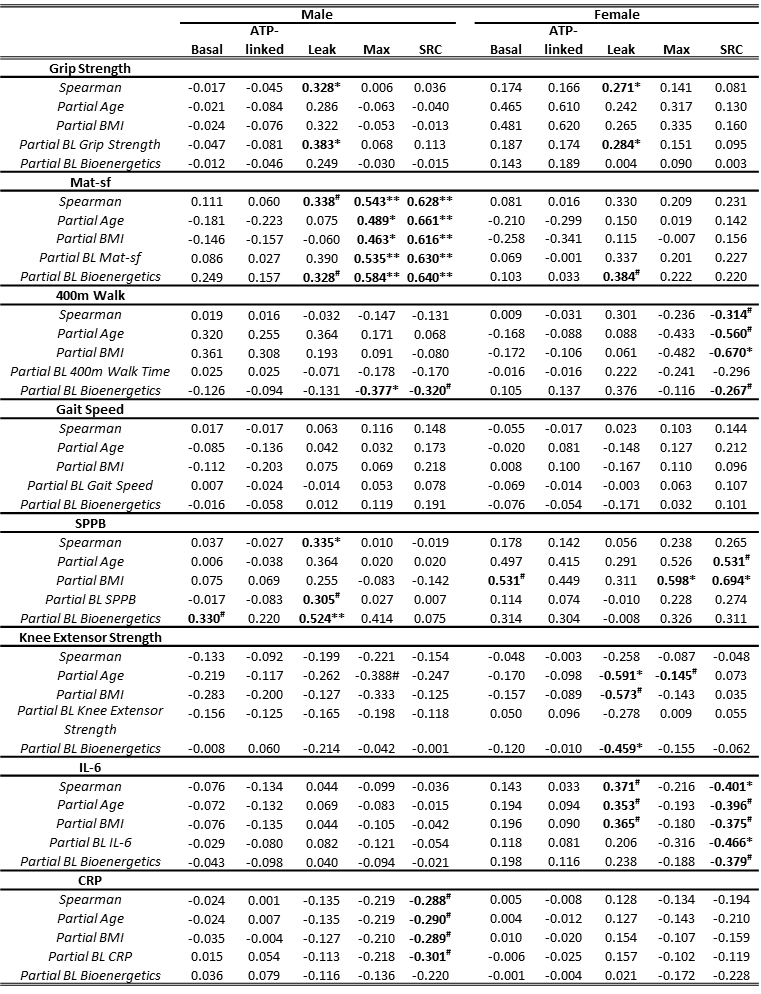
**

**Supplemental Table 5.** Parameters used for classification of responder status

| Change Mean ± SD | **RT** | **RT + CR** | **p-value for equality of variance** | **(RT ΔSD*1.15)** | **75% CI of random change** |
| --- | --- | --- | --- | --- | --- |
| Basal | 11.1 ± 23.1 | 3.4 ± 13.3 | 0.003 | 26.6 | -3.5, 49.7 |
| ATP-linked | 10.2 ± 19.8 | 1.9 ± 12.0 | 0.006 | 22.8 | -3.0, 42.6 |
| Leak | 0.5 ± 4.8 | 1.3 ± 3.0 | 0.008 | 5.5 | -0.7, 10.3 |
| Max | 23.1 ± 76.7 | 12.7 ± 43.4 | 0.002 | 88.2 | -11.5, 164.9 |
| SRC | 12.1 ± 57.4 | 9.3 ± 32.7 | 0.002 | 66.0 | -8.6, 123.4 |


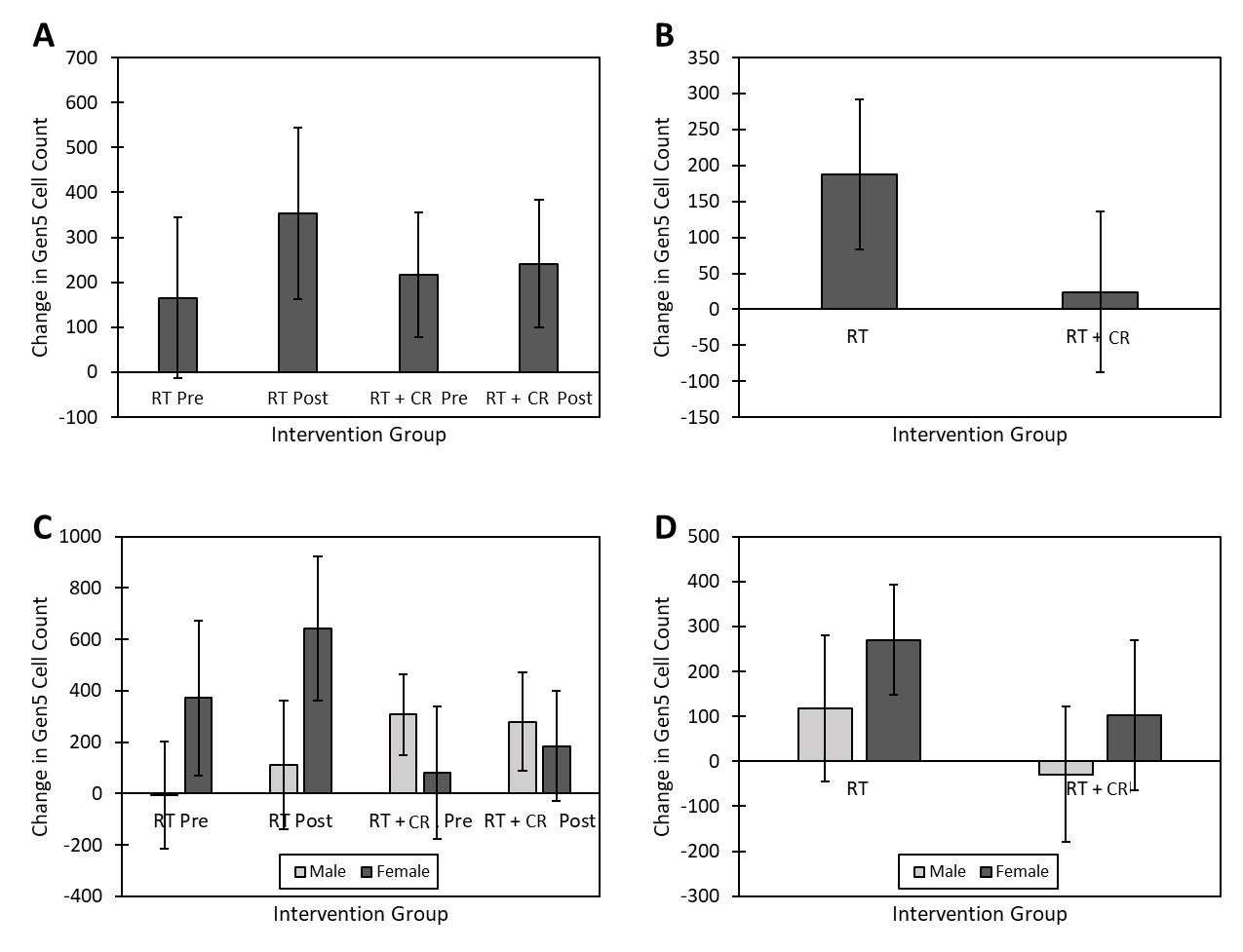


**Supplemental Figure 1. Changes in cell count after serum treatment**

1. Change in cell count pre- and post-intervention after serum treatment.
2. Change in cell count with intervention by intervention group (RT vs RT + CR).
3. Change in cell count pre- and post-intervention after serum treatment in both treatment groups by sex.
4. Change in cell count with intervention by intervention group and sex.
